# Biological engineering approaches for modulating the pathological microenvironment and promoting axonal regeneration after spinal cord injury

**DOI:** 10.3389/fnins.2025.1574763

**Published:** 2025-05-12

**Authors:** Xiaohong Chen, Rong Huang, Zhe Yang, Jun Zhang, Yanling Yang, Feng Gao, Minli Liu, Shengjun Zhang

**Affiliations:** ^1^Department of Pathology, Yan'an Medical College of Yan'an University, Yan'an, Shaanxi Province, China; ^2^Department of General Surgery, Affiliated Hospital of Yan’an University, Yan'an, Shaanxi Province, China

**Keywords:** spinal cord injury, regeneration of axons, pathological microenvironment, mitochondria, inflammatory response, glial scar, biological engineering

## Abstract

Functional recovery following spinal cord injury (SCI) presents significant challenges and imposes a substantial burden on society. Current research primarily focuses on minimizing damage and promoting regeneration to enhance functional recovery after SCI. Following SCI, secondary injuries such as mitochondrial dysfunction, vascular rupture, inflammatory responses, and glial scarring occur in the lesion area, forming the pathological microenvironment. These factors expand the extent of damage, exacerbate injury severity, and severely impede axonal regeneration after SCI. Modulating the pathological microenvironment through various interventions may facilitate axonal regeneration and promote functional recovery after SCI. This article reviews the influence and research advancements in axon regeneration concerning mitochondrial dysfunction, inflammatory response, and glial scar formation after SCI. Additionally, it integrates insights from bioengineering to improve the pathological microenvironment, summarizing the progress in axon regeneration research. The review concludes with novel strategies for enhancing axon regeneration, offering fresh perspectives for future investigations.

## 1 Introduction

Spinal cord injury (SCI) is a clinical syndrome caused by trauma, disease, or degenerative lesions, leading to structural and functional damage of the spinal cord and resulting in motor, sensory, and autonomic dysfunction below the level of injury. SCI can be categorized into two types: traumatic SCI, which results from accidents such as car crashes, falls from heights, and violent impacts; and non-traumatic SCI, which arises from conditions such as spinal cord tumors, spinal cord infarction, and degenerative diseases (e.g., cervical spondylotic myelopathy). According to the Global Burden of Disease Study (GBD), the prevalence and incidence of SCI have been increasing in recent years (Guan et al., 2023; Lu et al., 2025). Although SCI was once considered an “untreatable condition, “significant advancements in acute management and care have extended the life expectancy of SCI patients compared to the past. However, this also necessitates prolonged care and increases the risk of secondary complications, including neurogenic cystitis, respiratory disorders, and pressure ulcers (West and Cragg, 2024), thereby exacerbating the societal burden. To enhance functional recovery after SCI, it is essential to understand its pathophysiological mechanisms, which can be divided into primary and secondary injuries. Primary SCI involves direct mechanical insults, causing microstructural damage to spinal cord tissue and the formation of cystic cavities. Secondary injuries occur subsequently, leading to neuronal and glial cell death, mitochondrial dysfunction that impairs energy supply for axonal regeneration (Kim et al., 2024a,b), activation and aggregation of microglia and astrocytes at the injury site, triggering inflammatory responses, reactive gliosis, and the spread of the injury area, ultimately forming a pathological microenvironment unfavorable for regeneration. Furthermore, neurons lose their regenerative capacity during development, making axonal regeneration particularly challenging (Hilton et al., 2022; Akram et al., 2022). Given these factors, repair after SCI is limited due to developmental constraints, external microenvironmental changes induced by injury, and intrinsic biochemical alterations in neurons (Gwak et al., 2017). Therefore, improving the pathological microenvironment represents a promising strategy to promote axonal regeneration and functional recovery after SCI.

The repair of SCI primarily focuses on restoring motor and sensory functions as well as emotional regulation. Neural circuits play a critical role in modulating various physiological processes, including sensation, movement, cognition, and emotion. Axons are essential components of neural circuits, where neurons undergo polarization and develop growth cones that emerge from the cell body to connect with synaptic targets along the target direction, thereby forming complete neural circuits. Consequently, axon regeneration represents a key factor for functional recovery following SCI (Liu Y. et al., 2017; Li C. et al., 2024). Following SCI, structural damage disrupts neural circuits and causes stagnation of growth cones (Yao S. et al., 2024). Damaged central nervous system (CNS) axons form characteristic swellings at their tips, termed retraction balls, which indicate halted growth (Ertürk et al., 2007). Additionally, the expression of regeneration-associated genes decreases, diminishing the capacity to activate growth cones (Ko et al., 2016; Cheng et al., 2022). The presence of myelin debris and glial scars further impedes directed regeneration of synaptic connections with distant denervated targets (Wong et al., 2015; Pawar et al., 2015; Lai et al., 2022). To achieve axon regeneration, two strategies can be employed: enhancing intrinsic growth potential and improving the injury microenvironment. From an intrinsic growth perspective, the central domain of axons is rich in microtubules surrounded by actin filaments. Microtubules extend along the cytoskeleton toward the leading edge of growth cones, forming organized microtubule bundles (Santos et al., 2020). In contrast, retraction balls contain disordered microtubule networks. The stability of actin and microtubules determines whether the damaged axonal stump develops into a forward-moving growth cone or a non-growing retraction ball (Ertürk et al., 2007; Ducommun Priest et al., 2019; Zhang Y. et al., 2024). Pharmacological interventions or modulation of microtubule-associated proteins can facilitate axon regeneration post-injury (Pinto-Costa et al., 2020; Ribas et al., 2021). Furthermore, a layer of actin filaments exists at the interface between the central and peripheral domains. If these actin filaments become stagnant and aggregate, they will impede the extension of microtubules. Consequently, in the pathological microenvironment, promoting the division of the actin filament border (Pinto-Costa et al., 2020) represents a potential strategy for enhancing axon regeneration. Although certain studies have demonstrated that modulating specific axon-related transcription factors or proteins (Ma C. et al., 2023) can facilitate long-distance axon regeneration, from the perspective of the injury environment, as previously discussed, the damaged area is characterized by a series of secondary injuries, including mitochondrial dysfunction, inflammatory responses, and glial scarring, creating an adverse repair environment. Even with robust intrinsic growth capacity, rapid axon regeneration remains challenging under such conditions. In summary, to achieve axon regeneration, it is necessary to (1) re-activate developmental growth mechanisms and promote axonal sprouting (O'Donovan et al., 2014), and (2) eliminate factors inducing retrograde-like changes in growth cones, enabling regenerating axons to extend directionally through the injured region (Günther et al., 2015) and reconnect with target synapses.

Consequently, this paper elucidates the pathological microenvironment following SCI, summarizes the current therapeutic strategies targeting the microenvironment as well as advancements in bioengineering-based treatments, and establishes new directions for future axonal regeneration research.

## 2 Pathological microenvironment after spinal cord injury

The spinal cord is situated within the spinal canal and consists of gray matter surrounding the central canal and white matter located peripherally. It is enveloped by three layers of meninges, namely the dura mater, arachnoid mater, and pia mater, extending from the outermost to the innermost layer. Between these meningeal layers, there are deposits of fat, lymphatic vessels, venous plexuses, and spinal nerve roots, which are highly vascularized and play a critical role in providing structural protection and nutritional support to the spinal cord. When spinal cord tissue sustains injury, its structural integrity is compromised, leading to damage of nerves, blood vessels, and other components, thereby causing neurological dysfunction. Given the anatomical complexity of the spinal cord, implementing surgical interventions in clinical practice poses significant challenges, and ensuring consistent therapeutic outcomes remains difficult. Therefore, this article focuses on the internal microenvironmental changes induced by spinal cord injury and aims to identify potential breakthroughs for promoting post-injury repair.

**Figure 1:**
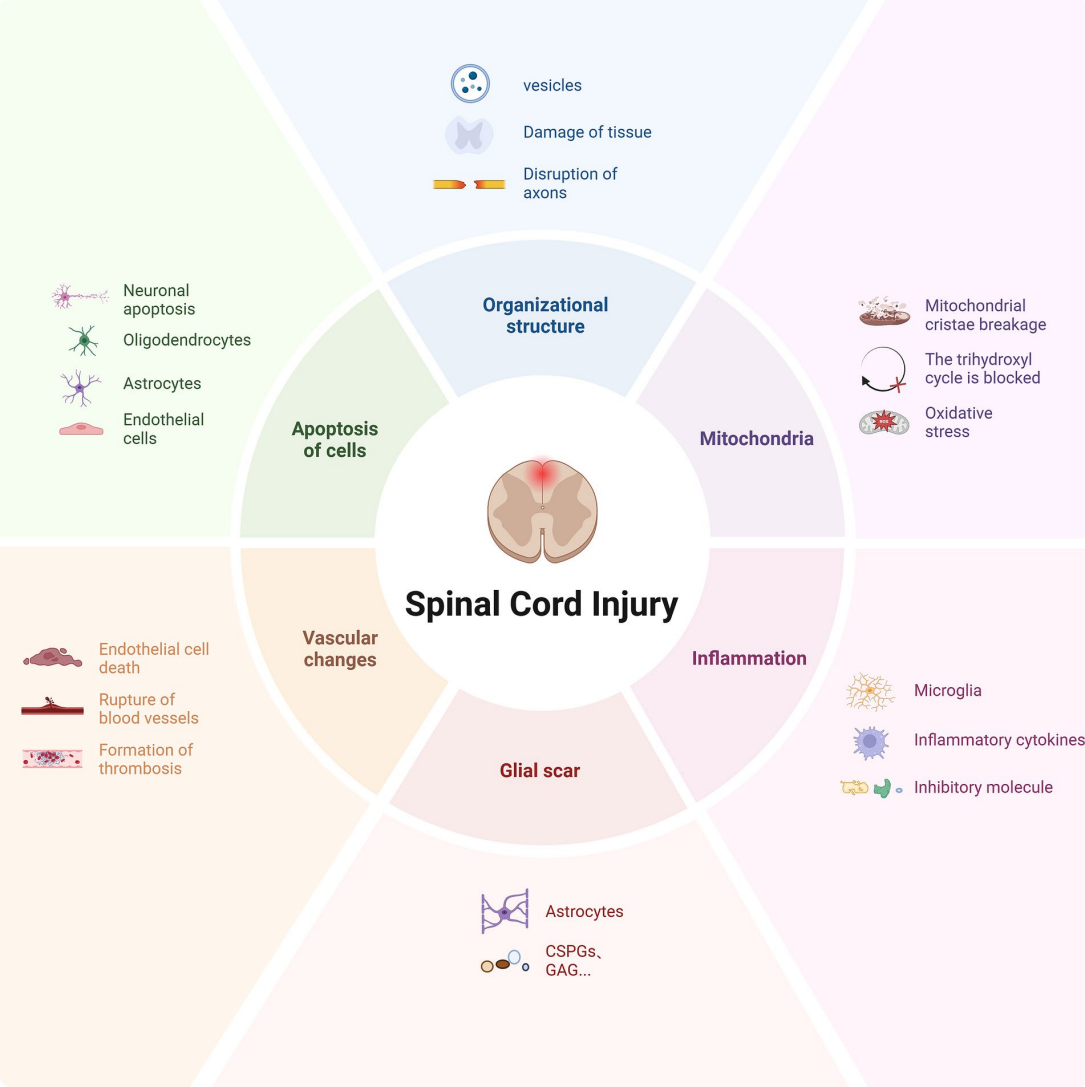
Pathological microenvironment after spinal cord injury. Following trauma, the spinal cord tissue structure undergoes significant changes, characterized by the formation of vesicles and vacuolization, leading to the disruption of axonal connections. Subsequently, a series of secondary injuries ensue, including mitochondrial dysfunction, inflammatory response, glial scar formation, insufficient blood supply, and apoptosis. These factors collectively contribute to the pathological microenvironment post-injury, which significantly hinders axonal regeneration.

When the spinal cord is impacted by various external factors, changes in the tissue and cytoskeleton structure are caused (He et al., 2016), resulting in fluid-filled cystic cavities and softening of the spinal cord tissue (Liu et al., 2020; Huang et al., 2024), which seriously hinders the growth cone of the damaged axon from extending through the injured area (He et al., 2016). Subsequently, it leads to pathological changes such as subcellular organelle (such as mitochondria) damage, cell dysfunction, chronic inflammation, and vascular changes. These changes activate astrocytes, microglia, fibroblasts, and other glial cells at the lesion site, and a large number of immune cells infiltrate, forming a dysfunctional pathological microenvironment (Kim et al., 2024a; Ma et al., 2024). The above various factors result in structural deficiency at the injury site and the formation of cystic cavities and scars (Ye et al., 2021), causing axonal side branches to bypass the injured area and reach the distal target neurons; in addition, the presence of myelin debris and myelin-associated growth inhibitory molecules (Poplawski et al., 2018), etc., cause the axon to change the growth direction or even stop growing (Figure 1). Therefore, the possible mechanisms driving axonal regeneration involve structural repair, provision and retention of nutritional factors, survival rate of progenitor cells, immune regulation, and other factors (Sun Z. et al., 2024).

### 2.1 Mitochondrial dysfunction in neurons after spinal cord injury

**Figure 2:**
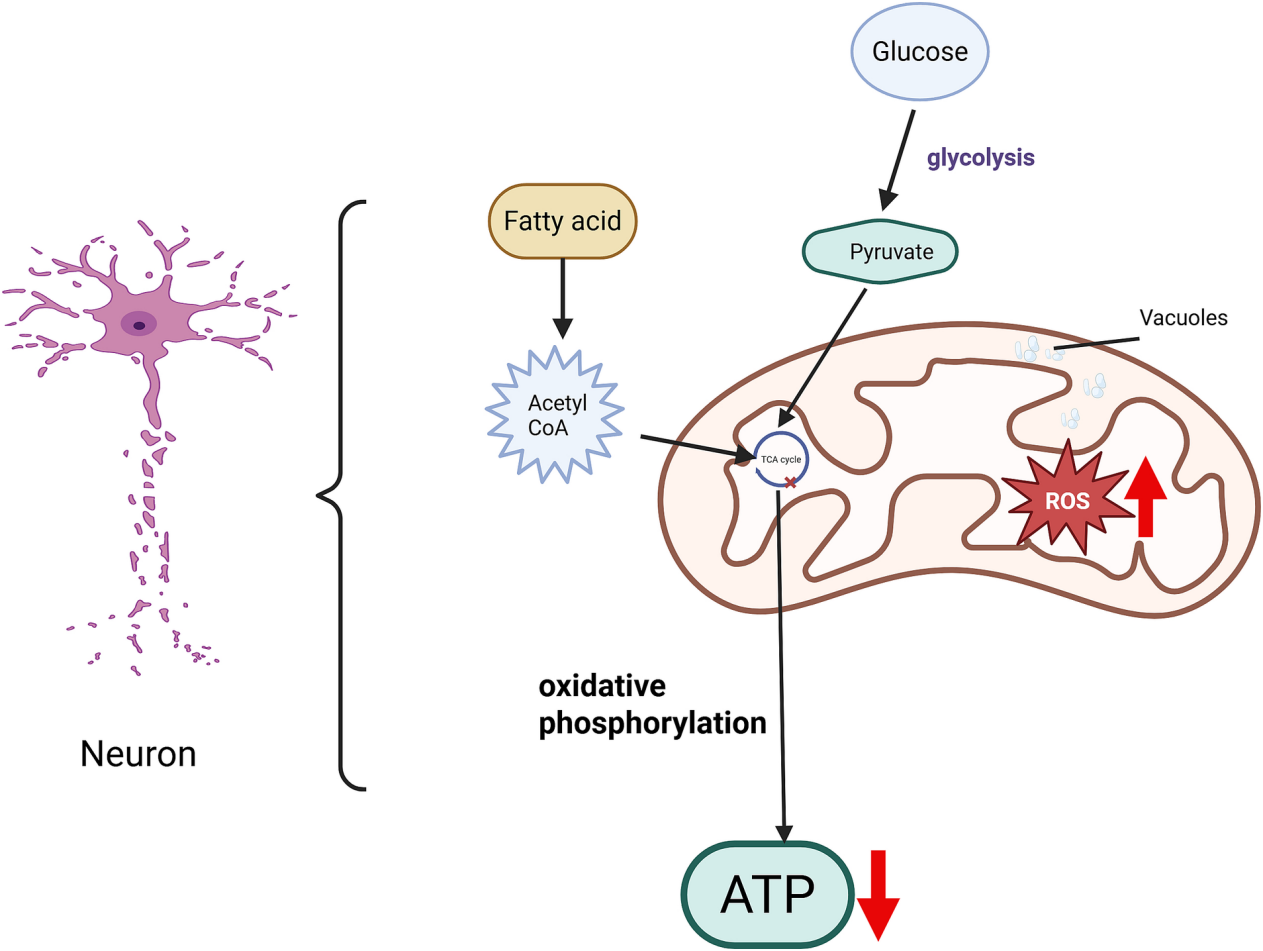
Mitochondrial dysfunction following spinal cord injury. Following spinal cord injury (SCI), the mitochondrial structure within neurons exhibits significant alterations. The orderly arrangement of mitochondrial cristae becomes disrupted, with a reduction in the number of cristae. Damage to both the inner and outer mitochondrial membranes leads to an expanded intermembrane space and the appearance of vacuoles. Such mitochondrial dysfunction impairs the tricarboxylic acid (TCA) cycle, consequently reducing energy production. Moreover, increased accumulation of reactive oxygen species (ROS) disrupts the balance of energy metabolism and the redox microenvironment. Collectively, these pathological changes hinder axonal regeneration and contribute to the limited recovery observed after SCI.

Mitochondria are the energy generation centers. For instance, oxidative phosphorylation that occurs on the inner mitochondrial membrane is the main process for energy generation and the main source of adenosine triphosphate (ATP) synthesis. The tricarboxylic acid cycle that takes place in the mitochondrial matrix can also release a small amount of energy, some of which is used for ATP synthesis. Therefore, mitochondria are the power sources of cells, and almost all cellular activities require ATP to provide energy. To ensure the continuous and stable transmission of neural signals, they are densely distributed in nerve cells and play a crucial role in neuronal development and axonal transport. However, after SCI, the arrangement of mitochondrial cristae is disrupted and their number decreases, the inner and outer membrane structures rupture, the intermembrane space becomes larger, a large number of vacuoles exist, causing mitochondrial swelling, resulting in the loss of matrix electron density and impaired mitochondrial function (Hassanzadeh et al., 2024), thereby hindering the processes of oxidative phosphorylation and tricarboxylic acid cycle and reducing ATP synthesis (Figure 2). After spinal cord injury, the axons need to re-seal the injured ends, rebuild the cytoskeleton, assemble the axonal components and form growth cones. These reconstruction processes require a large amount of energy, which needs to be provided by mitochondria in neurons (Han et al., 2020). Furthermore, there is a complex and close relationship between mitophagy and apoptosis. Under normal circumstances, mitophagy reduces the generation of apoptotic signals by eliminating damaged mitochondria; however, after spinal cord injury, the imbalance in the expression of mitochondrial fusion and fission genes (Hassanzadeh et al., 2024) and the limited function of mitophagy result in a large number of damaged mitochondria remaining at the injury site, exacerbating apoptosis, especially the apoptosis of neurons (Yao et al., 2023; Yao H. et al., 2024).

In addition, mitochondria are involved in the process of cell metabolism and signal transduction and are one of the main producers of reactive oxygen species (ROS). When mitochondria are damaged, the level of ROS will increase, leading to extensive disruption of the mitochondrial oxidative respiratory chain, imbalance of the redox microenvironment, and subsequent endothelial cell death. This cascade also results in blood-spinal cord barrier (BSCB) dysfunction and the prolonged presence of a pro-inflammatory microenvironment, causing a decrease in the vitality of neurons at the injury site, cell apoptosis, an imbalance in energy metabolism homeostasis, and a reduction in energy supply, which seriously hinders the regeneration of central axons (Ruan et al., 2023). Therefore, restoring the normal energy supply of mitochondria in neurons is the key to promoting axon regeneration.

### 2.2 Vascular system damage following spinal cord injury

During development, the vascular system plays a critical role in providing oxygen, nutrients, and hormonal signals, clearing metabolic waste from local tissues, and promoting cell circulation (Shi et al., 2024). Nerves and microvessels are distributed in a highly organized pattern throughout the interstitial tissues, ensuring optimal neural innervation and perfusion of the CNS, thereby supporting robust axonal growth. Structurally, the BSCB is formed through the endogenous fusion of endothelial cells (ECs) with tight junction (TJ) proteins and their interactions with astrocytes, pericytes, and perivascular microglia. It serves as a key interface between the spinal cord medulla and the peripheral vascular system, facilitating molecular exchange between circulating blood and the spinal cord to maintain CNS homeostasis (He et al., 2023; Gao P. et al., 2023). Acute mechanical compression of spinal cord tissue (Zhou et al., 2019) disrupts the tight junctions of endothelial cells, leading to microvascular rupture, BSCB damage, and leakage of plasma proteins, white blood cells, and other components. Inflammatory cells and factors from the periphery infiltrate the damaged area, inducing ischemic edema in the tissue (Bretheau et al., 2022; Ge et al., 2021). Within the first few minutes to weeks post-injury, processes such as cell death and inflammation are initiated, rapidly triggering a cascade of biochemical changes that culminate in progressive secondary injury and deprive lesioned cells of essential nutritional support (Bretheau et al., 2022; Yao et al., 2022). Therefore, emphasizing the repair of the BSCB after spinal cord injury by protecting specific cellular components or structural integrity is crucial to minimize subsequent damage (Gao P. et al., 2023).

### 2.3 Inflammatory response after spinal cord injury

**Figure 3:**
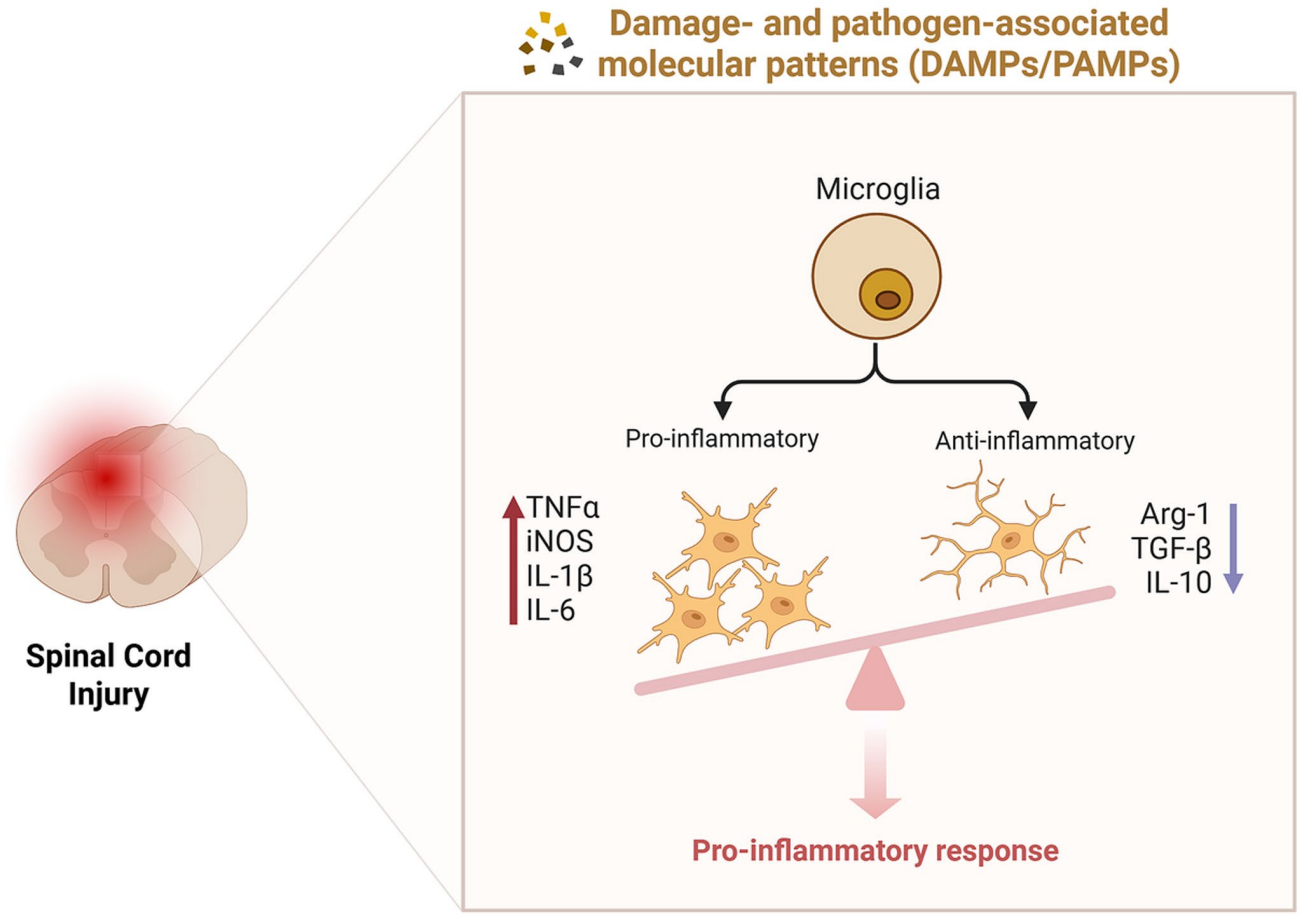
Inflammatory responses following spinal cord injury. The dynamic changes in microglial phenotypes significantly influence the inflammatory response. Following injury, environmental factors drive the predominance of pro-inflammatory microglia, which release pro-inflammatory cytokines (TNF-α, iNOS, IL-1β, IL-6), whereas anti-inflammatory microglia exhibit reduced production of anti-inflammatory cytokines (Arg1, TGF-β, IL-10). This imbalance results in the establishment of a pro-inflammatory microenvironment, exacerbating tissue damage and impeding repair processes.

Microglia, as resident macrophages within the nervous system, serve a critical role in immune surveillance. Following injury, microglia are influenced by pathological microenvironments such as vascular rupture, myelin debris, and NOD-, LRR- and pyrin domain-containing 3(NLRP3) inflammasome activation (Amo-Aparicio et al., 2022), leading to changes in their molecular phenotype, morphology, and functional properties (Paolicelli et al., 2022). These alterations activate a cascade of inflammatory signaling pathways. Initially, activated microglia exhibit protective characteristics; however, under the influence of the local environment, they rapidly transition into pro-inflammatory phenotypes, producing pro-inflammatory cytokines [Tumor necrosis factor-*α* (TNF-α), Interleukin-1β (IL-1β), Interleukin-6 (IL-6)] and ROS. These pro-inflammatory mediators further stimulate the infiltration of microglia, neutrophils, and exogenous macrophages, resulting in the release of additional inflammatory factors (Hellenbrand et al., 2021). In contrast, anti-inflammatory cytokines such as Arginase-1 (Arg1), Interleukin-4 (IL-4), Interleukin-10 (IL-10), and Interleukin-13 (IL-13) are present at low levels during the early stages of injury and exhibit limited anti-inflammatory effects (Hellenbrand et al., 2021; Figure 3). Pro-inflammatory mediators, including TNF-α, IL-1β, and IL-6, activate toll-like receptor 4 (TLR4) on microglia, which interacts with myeloid differentiation primary response gene 88 (MyD88; Rong et al., 2021), subsequently activating members of the nuclear factor κB (NF-κB) family (Wang et al., 2019). Additionally, ROS can trigger NF-κB signaling via the mitogen-activated protein kinase (MAPK) pathway (Liu et al., 2021), promoting the transcription of pro-inflammatory factors and exacerbating the inflammatory response. The presence of inflammation can further aggravate necrotic cell death, where undigested Deoxyribonucleic acid (DNA) from necrotic cells contributes to the formation of immune complexes, leading to the production of inflammatory cytokines and amplifying the inflammatory response (Nagata, 2018). This cascade ultimately results in widespread cell death, neuronal apoptosis, axonal damage, and demyelination (Song et al., 2024). Therefore, strategies aimed at reducing NLRP3 inflammasome activation (Jiao et al., 2020) and modulating inflammatory signaling pathways such as NF-κB (Su et al., 2019; Karova et al., 2019) may effectively control the inflammatory response following SCI, thereby promoting axonal regeneration post-injury (Song et al., 2019; Guan et al., 2024; Li X. et al., 2024).

### 2.4 Glial scar after spinal cord injury

**Figure 4:**
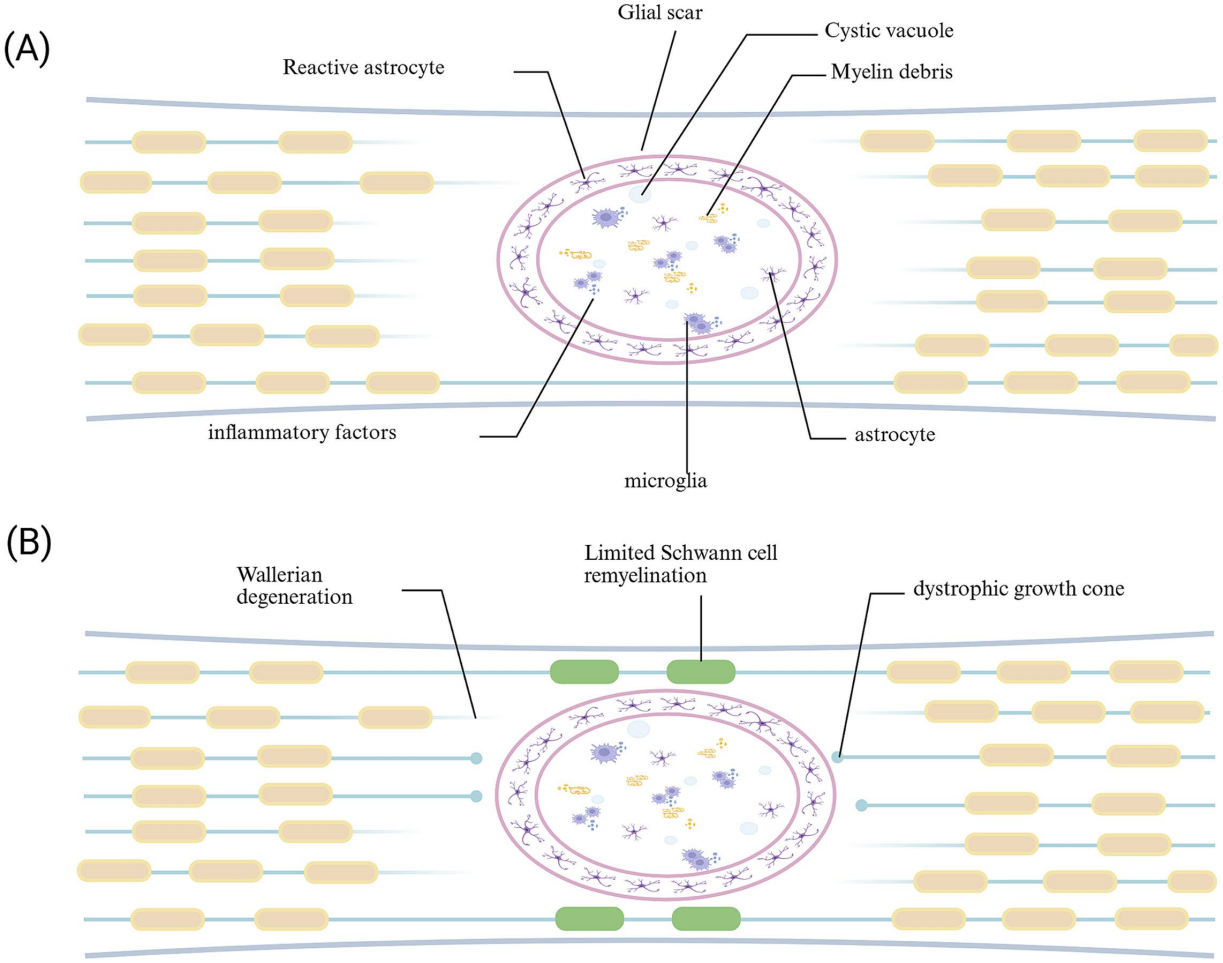
Glial scar following spinal cord injury. **(A)** During the acute phase, the formation of glial scar can limit the spread of injury. **(B)** In the chronic phase, the glial scar acts as a “barrier,” not only impeding the delivery of trophic factors to the injured area but also obstructing the passage and reconnection of regenerating axons with distant synapses.

Astrocytes are central nervous system supporting cells derived from neural progenitor cells, providing support, nutrition, and protection for neurons. Cell division is rarely observed in the CNS of healthy adults. When tissue damage occurs in the central nervous system, reactive astrocytes are proliferative and form a boundary around the damaged tissue (O'Shea et al., 2024). Studies have shown that when tissue damage occurs, original astrocytes (NAs) successively present different phenotypes. First are reactive astrocytes (RAs), which are divided into two phenotypes, neurotoxic and neuroprotective, according to the nature of the damage (Gottipati et al., 2020). At this time, reactive astrocytes are proliferative. They migrate to the lesion center and proliferate, forming a boundary around the damaged tissue, isolating inflammatory cells, preventing the spread of damage, and promoting tissue repair; meanwhile, it leads to an increase in the expression of chondroitin sulfate proteoglycan (CSPG; Gottipati et al., 2020), causing axon terminals to form retraction bulbs after contact with fibroblasts, stalling growth and severely hindering axon regeneration (Hu et al., 2024). Then there are scar-forming astrocytes (SAs; Hara et al., 2017). The long-term presence of glial scars hinders the passage of neurotrophic factors and impedes tissue repair (Figure 4). Therefore, understanding the intrinsic mechanism of reactive astrocyte phenotype transformation and controlling the duration of glial scar presence is crucial for axon regeneration.

## 3 By targeting the pathological microenvironment following spinal cord injury, we aim to enhance the capacity for axonal regeneration

After spinal cord injury, due to the destruction of tissue structure, mitochondrial dysfunction, incomplete vascular system, neuroinflammation and the existence of glial scars, axon regeneration is severely hindered. Therefore, in-depth understanding and regulation of the pathological microenvironment after SCI is of great significance for the later recovery.

### 3.1 Restoring mitochondrial function can help axon regeneration

Following SCI, the disruption of mitochondrial structure, in conjunction with the ischemic and hypoxic microenvironment, leads to impaired mitochondrial function. This disrupts the coupling between glycolysis and oxidative phosphorylation (Shi et al., 2024), thereby reducing energy production and supply. Axonal regeneration necessitates the mobilization of cellular resources, including proteins, lipids, and nucleotides, which impose significant energy demands (Yang and Zhou, 2025). Consequently, it is essential to adopt a dual strategy: promoting the recovery of mitochondrial structure to enhance energy production, while simultaneously regulating the detrimental feedback loop between the microenvironment and mitochondrial function. Such an approach facilitates enhanced mitochondrial transport, providing the necessary energy for axonal regeneration (Han et al., 2020; Wang et al., 2018).

Cardiolipin (CL) is a unique mitochondrial phospholipid located exclusively in the inner mitochondrial membrane (IMM) and plays a critical role in mitochondrial bioenergetics. Following spinal cord injury, restoring alterations in cardiolipin levels and preserving mitochondrial morphology and structure can mitigate mitochondrial dysfunction (Liu N. K. et al., 2022). Lactate, as a by-product of glycolytic metabolism under hypoxic conditions, is also an energy substrate and signaling molecule, which is crucial for metabolic flexibility and axonal regeneration after SCI (Shi et al., 2024). However, the expression of monocarboxylate transporter 1 (Monocarboxylate transporter 1, MCT1) in endothelial cells after SCI decreases, preventing lactate from being transported into neurons, resulting in metabolic imbalance and hindering axonal regeneration (Shi et al., 2024). Exogenous and endogenous lactic acid administration can promote histone lactylation, modulate metabolic reprogramming, and facilitate axonal regeneration (Hu et al., 2024; Li et al., 2020). Following SCI, damaged axons exhibit the involvement of syntaphilin (Snph), a static anchor protein that halts mitochondrial transport in growth cones by interacting with microtubules (Han et al., 2020). Inhibition of Snph-mediated anchoring enhances axonal mitochondrial transport, enabling the removal of damaged mitochondria and replenishment with healthy ones, thereby fulfilling the elevated energy requirements during the regeneration process.

Furthermore, transplantation of bone marrow mesenchymal stem cells (BMSCs) into the injured area facilitates the transfer of mitochondria from BMSCs to damaged neurons, thereby enhancing ATP production and calcium buffering capacity (Li et al., 2019; Li et al., 2021). Additionally, the peptide sequence cations-cysteine-alanine-glutamine-lysine (CAQK) can be conjugated with mitochondria to form a mitochondrial compound. This compound is delivered via intravenous administration and specifically targets macrophages within the injured region, promoting the phagocytosis of inhibitory myelin debris. However, excessive phagocytosis may result in lipid accumulation and disruption of intracellular lipid homeostasis, which could impede axonal regeneration (Xu et al., 2024b). Consequently, further experimental validation is required to determine the optimal timing for mitochondrial supplementation to maximize its therapeutic effects.

In conclusion, the restoration of normal mitochondrial function is advantageous for axonal regeneration following SCI. Nevertheless, further investigation is required to validate both the temporal dynamics of its effects and the underlying mechanisms.

### 3.2 Improving the vascular system helps to regenerate axons

After spinal cord injury, the lateral connections of endothelial cells are disrupted, indicating impaired vascular integrity, which triggers the disruption of the BSCB, activation of immune cells, metabolic disorders, lipid peroxidation, and accumulation of neurotoxic substances (Gao P. et al., 2023; Xu et al., 2024a), and also induces endothelial cell death, resulting in incomplete formation of the vascular network. It has been shown that endothelial cells play a critical role in maintaining the integrity of vascular networks (Li et al., 2024a). Consequently, vascular reconstruction can be approached from both endogenous and exogenous perspectives to ensure the preservation of BSCB integrity.

The upregulation of endogenous osteopontin (OPN), α5β1 integrin, and Sirtuin 1 (SIRT1; Jiang et al., 2023; Xu et al., 2024a; Halder et al., 2018), as well as the inhibition of the JAK1/STAT3 signaling pathway (Gao P. et al., 2023), can modulate endothelial barrier function. Additionally, exogenous administration of ginsenoside Rg1 (Yin et al., 2024) and other pharmacological agents further promotes angiogenesis in the spinal cord. However, blood vessels formed by endogenous endothelial cells are typically leaky, insufficient for complete reconstruction of the BSCB, and incapable of providing adequate metabolic support locally (Xu et al., 2024a). Furthermore, during the chronic phase, dense fibrotic scar formation, excessive extracellular matrix (ECM) deposition, and the inability of glial cells and axons to regenerate result in an incomplete vascular network (You et al., 2023). Early intervention is critical to promote timely angiogenesis. Mesenchymal stem cells exhibit angiogenic potential, and certain subpopulations of exosomes possess the ability to stabilize the BSCB. Consequently, hydrogel-mediated transplantation of stem cells or combination with exosomes may induce robust vascular growth, promote neuronal differentiation, and supply essential nutrients to the injured area (Li L. et al., 2022; Guo et al., 2020).

### 3.3 Reducing inflammation helps to regenerate axons

Initially, neuroinflammation serves as a defense mechanism that can phagocytose harmful substances such as tissue debris and necrotic cells, helping with tissue repair; however, the excessive phagocytic activity of reactive microglia and macrophages can result in lipid accumulation and disruption of intracellular lipid homeostasis, subsequently eliciting an inflammatory response (Xu et al., 2024b). Additionally, the phagocytic ability is limited. Eventually, inhibitory substances such as myelin debris will accumulate in large quantities, and the inflammatory response persists for a long time, causing severe secondary damage and hindering the recovery of neural function (Li X. et al., 2024). Therefore, reducing the pro-inflammatory response and enhancing the phagocytosis of inhibitory components such as myelin debris will create a more permissive environment for axonal regeneration (Lemmens et al., 2015).

Regarding the phenotypic changes of microglia, on the one hand, Li et al. developed reactive composite hydrogels loaded with MG53 protein. These hydrogels alleviated neuroinflammation through the inhibition of pro-inflammatory microglia and facilitated both neurogenesis and axonal regeneration (Li X. et al., 2024). On the other hand, the implantation of PDA nanoparticles integrated with anisotropic scaffolds loaded with glial-cell-line-derived neurotrophic factor (GDNF; Ma J. et al., 2023) or CS-CA-DA hydrogels (Liu K. et al., 2022), can facilitate the polarization of microglia toward an anti-inflammatory phenotype, augment their phagocytic capacity for myelin debris clearance, restore lipid metabolic homeostasis (Chen et al., 2024) and inhibit local inflammatory responses. To enhance the phagocytic capacity, direct transplantation of anti-inflammatory microglia can be employed to modulate the inflammatory milieu following spinal cord injury, thereby promoting neural regeneration and restoring homeostasis within the spinal cord (Guan et al., 2024). Microglia is beneficial for the repair after spinal cord injury because neuroinflammation can clear the damaged tissue, secrete cytokines, and promote regeneration (Wang et al., 2022b). This is intricately associated with the anti-inflammatory properties of microglia. Therefore, it requires further experimental exploration to determine the timing of the phenotypic transformation of microglia or which phenotype is dominant when both phenotypes coexist.

In addition, after inflammation is activated, a large amount of ROS is released, forming a damaging microenvironment where neuroinflammation and oxidative stress coexist, which seriously hinders axonal regeneration. The regenerative microenvironment can be reconstructed by scavenging ROS, regulating cytokines or administering drugs (Park et al., 2018; Li et al., 2023). For example, administration of low-concentration paclitaxel can weaken the upregulation of inhibitory molecules after spinal cord injury and promote axonal extension (Roman et al., 2016).

### 3.4 Regulating the formation of glial scar is helpful for axon regenerate

After SCI, the ECM within the glial scar secretes inhibitory factors, such as CSPGs and glycosaminoglycans (GAGs; Naderi et al., 2018), which suppress axonal growth. Upon contact with the proteoglycan-rich substrate, the growth of the axon tip ceases (Huang et al., 2024; Hu et al., 2024; Varadarajan et al., 2022), thereby further restricting the potential for axonal regeneration (Wu et al., 2017). The research by Hee Hwan Park et al. shows that the I-5 hydrogel forms a complex with ARSB (a human enzyme that degrades CSPG) through hydrophobic interaction, which can significantly reduce fibrotic ECM components to alleviate the fibrotic microenvironment (Park et al., 2022). Therefore, changing mechanical signals, softening scars, and reconstructing the natural projections of characteristic neurons are important components of the axon regeneration strategy aimed at restoring lost neural functions (Squair et al., 2023). For instance, Huang et al. upregulated the expression of Fastin-1, significantly inhibiting the level of active myosin in microglia, achieving the goal of reducing the stiffness of the injured spinal cord, promoting the migration and differentiation of neurons at the injury site, facilitating axon regeneration, and aiding functional recovery (Huang et al., 2024). Additionally, the activation of endogenous neurogenesis through bioactive materials can restore sensory/motor functions after complete SCI by forming new relay neural circuits (Hao et al., 2023). Zhao et al. first trimmed the scars and cystic structures and then injected NT3 chitosan to achieve the repair of SCI in the chronic phase (Zhao et al., 2022). In mouse models of spinal cord injury, pharmacological blockade of the interaction of type I collagen in reactive astrocytes can prevent astrocyte scar formation, thereby improving axon regeneration and achieving better functional outcomes (Hara et al., 2017). Reducing NB-3 in glial scar-forming cells can promote axon regeneration across the glial scar through the injured area and achieve long-distance regeneration (Huang et al., 2016).

Besides, since glial scars have both advantages and disadvantages, a thorough understanding of the timing of astrocyte scar formation is of great significance for promoting directed axonal regeneration. It has been reported that preventing reactive astrocytes from forming glial scars leads to extensive infiltration of the injured site by inflammatory cells, increased neuronal loss, severe tissue degeneration, and ultimately failure of spontaneous functional recovery (Gu et al., 2019). It has been reported that Mg2 + released by MgO alleviates apoptosis by blocking calcium influx, and PUR/RA promotes the recruitment and neuronal differentiation of endogenous NSCs. Implanting MgO/PLCL scaffolds loaded with PUR/RA in the chronic phase of SCI can reduce glial scar formation at the SCI injury site and promote axonal regeneration (Xie et al., 2022).

It is reported that platelet-derived growth factor receptor *β* (PDGFRβ) is a biomarker of fibrotic scar-forming fibroblasts. After spinal cord injury, the expressions of PDGFD (secreted by astrocytes) and PDGFB (secreted by macrophages/microglia and fibroblasts) increase, which can activate PDGFRβ and promote the formation of fibrotic scars (Li Z. et al., 2022); Implantation containing miR-26a can inhibit the PTEN and GSK-3β signaling pathways in neurons, and make neurites elongate directionally to promote neuronal reconnection and functional recovery (Gao X. et al., 2023). Therefore, understanding the stimulatory factors for the formation of fibrotic scars and the inhibitory molecules in the fibrotic environment is of the utmost importance for promoting axonal regeneration.

## 4 Advances in promoting axon regenerate after injury in stem cell transplantation and tissue engineering

The repair of SCI is critically dependent on microenvironment remodeling and the promotion of endogenous stem/progenitor cell recruitment and neuronal differentiation (Xu et al., 2021). Recent research has primarily concentrated on two key strategies to achieve these objectives. Firstly, regenerative medicine has progressed through several stages: from direct stem cell transplantation to the development of bioactive scaffolds for cellular delivery, and further to the integration of bioengineering techniques that concurrently transplant growth factors, drugs, and cells to induce neural tissue regeneration and motor function recovery. Secondly, rehabilitation medicine leverages the intrinsic plasticity of the CNS, employing methods such as exercise training, electrical stimulation, neurochemical modulation, and combined dual-stimulation approaches to retrain uninjured neural pathways (Yao S. et al., 2024), thereby establishing new neural circuits and achieving functional restoration. Despite these advancements, the inherent complexity of the CNS presents ongoing challenges for various repair strategies. Consequently, this review proposes novel therapeutic approaches aimed at enhancing axonal regeneration.

### 4.1 Research advances of regenerative medicine in axon regeneration

#### 4.1.1 To repair the damaged tissue structure in the field of bioengineering

SCI results in structural damage and alterations to the neural tissue components, leading to retraction and cessation of axonal growth cones. Growth cones play a critical role in guiding the direction of axonal regeneration; however, following SCI, the collateral branches of growth cones at the injury margin often bypass the lesion area and extend toward distal target neurons (Varadarajan et al., 2022). Therefore, implementing bridging strategies to provide a physical substrate for axonal regeneration is essential (Novikova et al., 2018). Researchers have employed various materials such as extracellular matrices, hydrogels, and biological scaffolds within the injury site to fill cavities and mimic the composition and microstructure of spinal cord tissue. This approach facilitates the regeneration of axonal collaterals through the injury zone and promotes recovery of the affected region. Moreover, these implanted materials exhibit biocompatibility with internal tissues and work synergistically to provide structural support, regulate signal transduction (Sun Z. et al., 2024; Ke et al., 2023), and significantly influence cell proliferation, migration, and differentiation (Yao S. et al., 2024; Li X. et al., 2024; Qiu et al., 2024). Consequently, these materials effectively enhance the regenerative microenvironment post-SCI, thereby promoting axonal regeneration. Among these, the ECM is a dynamic network structure (Qiu et al., 2024), primarily composed of collagen, proteoglycans, fibronectin, and cytokines. Studies have shown that ECM during early developmental stages effectively guides tissue regeneration in adults (Sun Z. et al., 2024). Fan et al. developed a natural ECM-based biopolymer, hyaluronic acid hydrogel, which, when implanted at the injury site, accelerates myelin regeneration, axonal regeneration, and angiogenesis (Fan et al., 2024). Consequently, this material has found widespread application in biomaterials. Hydrogels can mimic the mechanical properties of soft tissues (Yao S. et al., 2024) and the physiological state of the ECM (Zhang W. et al., 2024). By filling cavities through structural substitution, they facilitate cell adhesion, expansion, and differentiation at the injury site, thereby promoting nerve regeneration and functional recovery while alleviating the inflammatory microenvironment and reducing glial scar formation.

Research indicates that the Kiet A. Tran team described a method of modifying Digital Light Processing (DLP) to print scaffolds that can replicate the varying proportions of white and gray matter in spinal cord tissue (Tran et al., 2023). By utilizing solid 3D gelatin microsphere (GM) scaffolds, they successfully bridged the injury gap (Ke et al., 2023). With advancements in biomaterial research, biomaterials are now capable of not only mimicking structural features but also replicating tissue functions. For instance, DPMSCs have been assembled into a biological construct called Spinor, which not only exhibits geometric characteristics similar to those of spinal cord tissue but also autonomously releases exosomes with optimal quantity and quality to inhibit scarring and inflammation and promote axonal regeneration (Yan et al., 2022). Additionally, studies have found that excessive phosphorylation of tau, a microtubule-associated protein abundant in neuronal axons, can destabilize microtubule bundles. This instability and disorder can cause active growth cones to become malnourished and cease growth. To address this issue, the authors combined the antioxidant idebenone with the microtubule stabilizer paclitaxel and formulated a nano-drug by incorporating it with chondroitin sulfate proteoglycan. This formulation allows the drug to remain in the spinal cord for at least 2 weeks, stabilizing the microtubule structure and environment, thereby significantly enhancing hindlimb motor function and promoting axonal regeneration (Xu et al., 2024).

However, the injured area experiences substantial deposition of inhibitory factors and significant loss of trophic molecules. Simply restoring tissue structure and establishing connections is insufficient to fully promote axonal regeneration, as the released nutrients alone cannot adequately stimulate this process. Moreover, while there is an inherent capacity for spontaneous recovery following injury, studies have shown that the neural mechanisms by which functional biological scaffolds improve motor outcomes primarily rely on the formation of neuronal bridging. This approach does not facilitate long-distance regeneration of descending axons across the entire injury gap and may even compromise the intrinsic spontaneous recovery ability (Liu et al., 2019). Therefore, further research and experimentation are required to address the retention time following structural repair, the release and retention of trophic factors, and the overall improvement of the injury microenvironment.

#### 4.1.2 Cell transplantation promotes neuronal differentiation

In basic research, transplanting Schwann cells, neural stem cells or progenitor cells, olfactory ensheathing cells (OECs), oligodendrocyte precursor cells, and mesenchymal stem cells (MSCs) into the injured area to construct a complete neural network creates a favorable microenvironment for axonal regeneration and the survival and synaptogenesis of NSC-derived neurons (Lai et al., 2016). This approach facilitates their differentiation into functional motor neurons within the injured region (Kim et al., 2024a; Song et al., 2024). Moreover, multiple studies have demonstrated that cell transplantation is an effective therapeutic strategy for repairing SCI. For instance, Wang et al. found that transplanting neural progenitor cells (NPCs) derived from human embryonic stem cells can promote the regeneration of serotonergic axons (Wang et al., 2022a). Human umbilical cord blood-derived MSCs (hUCB-MSCs) reduce astrocyte activation and enhance axon preservation (Yang et al., 2018). Transplanting sheets of adipose-derived mesenchymal stem cells (ADSCs) has been shown to be more effective than single-cell transplantation in reducing tissue fibrosis (Chen et al., 2022). Additionally, ADSCF loaded with NPCs improves the survival, maturation, axonal regeneration, and motor function of SCI rats (Chen et al., 2023).

Growing axons are either covered by myelin sheaths or encapsulated by endogenous Schwann cells, which migrate to the lesion site and extend the survival duration (Chedly et al., 2017). In addition to direct stem cell transplantation, utilizing extracellular vesicles (EVs) derived from MSCs as an alternative to MSC transplantation can mimic paracrine signaling and offers a promising strategy for modulating the microenvironment (Wang et al., 2021).

#### 4.1.3 Cell combined with biomaterial transplantation promotes axon regeneration

**Figure 5:**
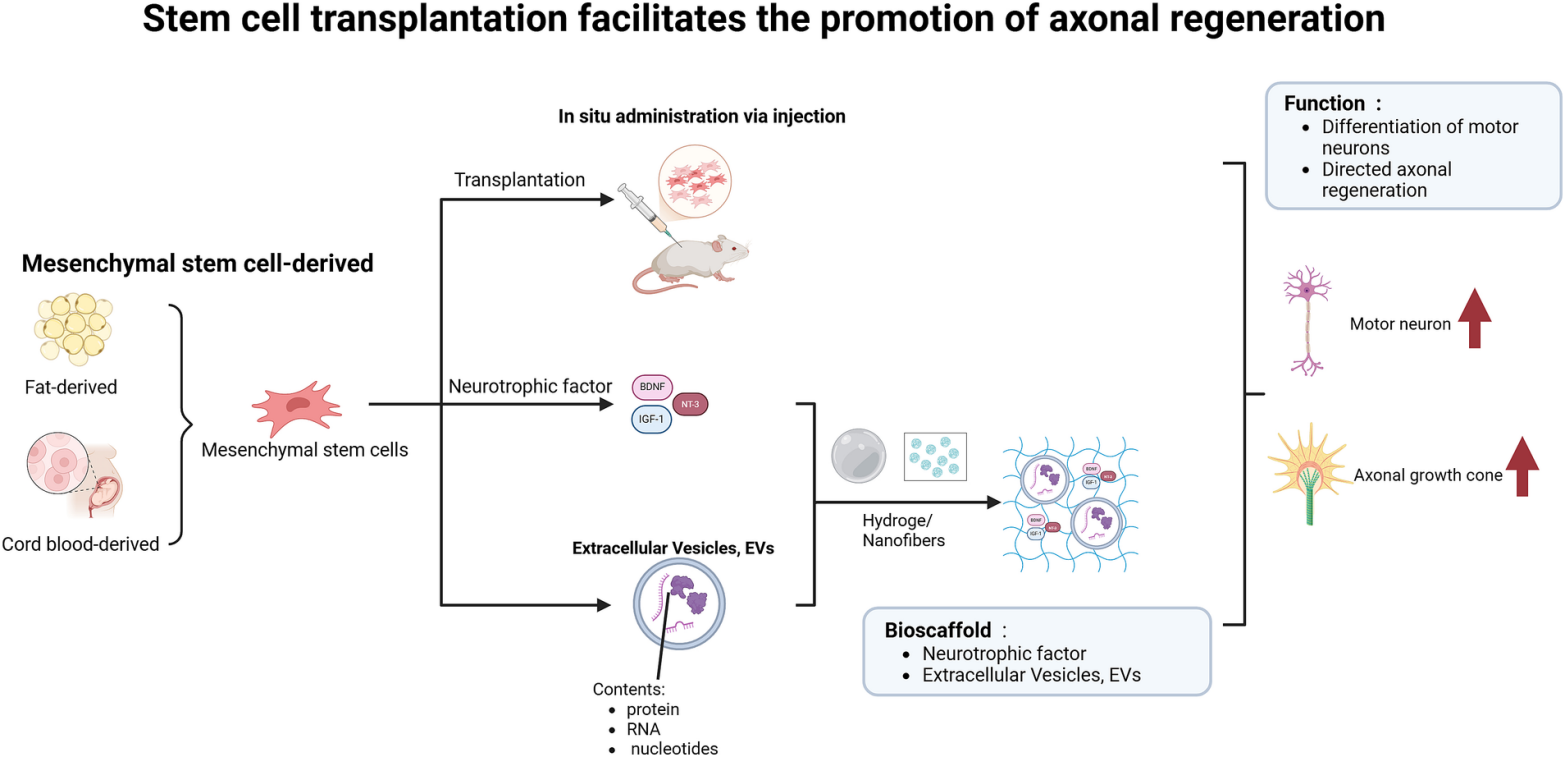
Stem cell transplantation facilitates axonal regeneration and functional recovery in spinal cord injury models. The implantation of adipose-derived or umbilical cord blood-derived mesenchymal stem cells into the injured area, along with the release of neurotrophic factors, facilitates the differentiation of functional neurons. Furthermore, when combined with biomaterials such as hydrogels and nanofibers, this approach enables targeted delivery of nutrients and enhances axonal regeneration.

Stem cell transplantation, which can differentiate into neurons, has a positive impact on the repair of SCI and addresses the issue of insufficient endogenous neural stem cells (NSCs) following injury (Figure 5). Studies have demonstrated that the combined transplantation of MSCs engineered to overexpress brain-derived neurotrophic factor (BDNF) and induced pluripotent stem cell-derived motor neuron progenitor cells (iMNPs) not only compensates for deficiencies in neurotrophic factors but also directs the differentiation of mature motor neurons in the injured spinal cord, thereby promoting axonal regeneration (Kim et al., 2024a). However, some reports indicate that unfavorable microenvironments can lead to uncontrolled differentiation of transplanted cells (Fan et al., 2018), such as NSCs predominantly differentiating into astrocytes rather than neurons, which exacerbates glial scar formation (Li et al., 2018). Additionally, the ischemic environment hinders the stable proliferation of endogenous NSCs *in vivo*, leading to short retention times and low survival rates (Zhang W. et al., 2024), thereby limiting the regenerative capacity of these cells (Führmann et al., 2017). There is an urgent need to develop a delivery system for NSCs that can mitigate the inhibitory microenvironment following SCI, ensuring stable proliferation and directed differentiation of NSCs into functional neurons (Zhang W. et al., 2024). Experimental evidence demonstrates that combining biodegradable hydrogels with neural stem/progenitor cells (NSPCs) can create a more permissive environment for the survival and integration of transplanted cells (Silva et al., 2017). The authors utilized highly aligned poly (glycolide) nanofibers, which reduce astrocyte reactivity, promote extensive directional mixing between Schwann cells (SCs) and astrocytes (ACs), and ultimately facilitate the growth of numerous neurites from the SC compartment into the AC region (Achenbach et al., 2023). Additionally, to further enhance the survival rate of transplanted cells, MSCs can be cultured to form sheet-like structures and subsequently processed into decellularized extracellular matrix (dECM) for implantation at the injury site, thereby promoting axonal regeneration and functional recovery (Qiu et al., 2024).

#### 4.1.4 Multi-modal integration for the targeted delivery of nutrients

Due to the limited axonal regeneration capacity following SCI and the disconnection of neural elements, as well as the restricted systemic drug delivery efficiency caused by the blood-spinal cord barrier, developing effective therapeutic strategies for SCI in clinical practice remains highly challenging (Ran et al., 2023). Although biomaterials such as hydrogels can provide a favorable microenvironment for the survival, proliferation, and differentiation of transplanted cells, the lack of essential nutritional factors limits their biological potential (Song et al., 2024). For instance, BDNF not only promotes the neural differentiation of stem cells but also facilitates the regeneration of damaged axons (Butenschön et al., 2016). Therefore, the synergistic interaction among biomaterials, cell transplantation, and growth factors can guide axons through the lesion site, provide a supportive cellular matrix, stimulate axon growth, and extend the growth distance of regenerating axons (Liu S. et al., 2017). Continuous delivery of neurotrophic factors is critical for establishing a microenvironment conducive to cell survival and nerve regeneration in SCI (Li et al., 2016). For instance, Song et al. implanted an IGF-1 (Insulin-like Growth Factor 1) gel containing NSCs at the injury site, which significantly promoted neurite outgrowth and myelin regeneration, thereby enhancing neural recovery following SCI (Song et al., 2024). Researchers developed a biological scaffold based on autologous plasma exosomes (AP-EXO), loaded with neuronal-targeting peptides and growth-promoting peptides. This scaffold can be specifically delivered to neurons in the injured area, triggering robust axonal regeneration at the injury core and reconstructing intraspinal circuits, thus promoting motor function recovery in mice after SCI (Ran et al., 2023). Additionally, grafts carrying a half-dose of SOX9 were found to promote the differentiation of NSCs into motor neurons, reduce glial scar matrix accumulation, facilitate long-distance axonal growth, and establish connections with distal target neurons, leading to significant improvements in both motor and sensory functions in recipient animals (Liu et al., 2023). Furthermore, the recovery of the injured area is not solely dependent on the supplementation of a single nutrient. For instance, EVs can carry multiple bioactive substances, including lipids, miRNAs, proteins, and nucleic acids, delivering diverse nutrients to the injury site. By incorporating EVs into hydrogels, which can remain in the tissue for several weeks, long-term anti-inflammatory effects are sustained, thereby promoting axonal regeneration (Wang et al., 2023). Integrating exosomes from subpopulations of bone marrow mesenchymal stem cells (CD271 + CD56 + BMSC-Exos) into hydrogels and implanting them *in situ* can enhance the expression of neurofilament (NF) and synaptophysin (Sun Y. et al., 2024). Additionally, certain biomaterials can induce the release of growth factors and other bioactive molecules. For example, a biomimetic hydrogel scaffold coated with hyaluronic acid/silk fibroin/polydopamine achieved localized and sustained release of neurotrophic factor-3 (NT-3; Sha et al., 2023). *In situ* injection of HSP-F/BCS hydrogels can inhibit glial scar progression, reduce microglia/macrophage infiltration, promote angiogenesis, and induce myelinated axonal regeneration (Sorouri et al., 2023).

The aforementioned studies have demonstrated that the implantation of biological materials and related nutritional formulations can stimulate axonal regeneration, synaptogenesis, and angiogenesis, thereby reducing the size of the injury and significantly improving motor function recovery in SCI rats. However, without appropriate guidance, axons rarely extend through the lesion site (Günther et al., 2015). Therefore, further research is needed to explore how to guide axons to grow directionally and to determine whether regenerated axons reconnect with inactive synapses or establish new synaptic connections. Additionally, the mechanisms underlying the directional guidance of axonal growth require further experimental validation.

### 4.2 Research progress on the combination of regenerative medicine and rehabilitation therapy in axon regeneration

Regenerative medicine leverages biomaterials to bridge the damaged area and facilitates targeted delivery of neural progenitor cells, drugs, neurotrophic factors, and other therapeutic agents. This approach can improve the pathological microenvironment at the injury site, thereby promoting axonal regeneration. Additionally, rehabilitation therapy encompasses two key strategies: first, stimulating neural circuits through exercise to enhance neural conduction efficiency; second, employing physical therapies such as electrical and magnetic stimulation to activate neuronal activity via electrical and mechanical signals. Therefore, for optimal repair following SCI, it is essential to integrate regenerative medicine with rehabilitation therapy. By leveraging neural plasticity, re-stimulating neural circuits can maximize functional recovery of newly regenerated axons (Yao S. et al., 2024).

#### 4.2.1 Advancements in the research of electrical stimulation in conjunction with biomaterials for axon regeneration

Following SCI, sensory and motor functions are impaired, and neural circuits become inactive. Electrical stimulation can enhance neuronal activity post-injury (Hutson et al., 2019). Epidural electrical stimulation and muscle stimulation can mimic the feedforward and feedback electrical signals in the spinal cord’s sensory-motor circuits, thereby activating genes associated with axonal regeneration of motor neurons (Zhou et al., 2024), which promotes axonal regeneration. Conductive hydrogels significantly improve SCI repair efficiency by restoring interrupted electrical signals along the spinal cord conduction pathways. For instance, conductive hydrogels loaded with M2-EXOs not only stimulate the differentiation and axonal growth of neural stem cells in the dorsal root ganglia and modulate the phenotypic expression of microglia (Guan et al., 2024), but also inhibit astrocyte differentiation (Luo et al., 2022). Furthermore, axons must regenerate directionally through the damaged area. To this end, directional conductive hydrogel fibers with an orderly-aligned structure have been developed. These fibers provide immediate electrical connections and direct the transmission of electrochemical signals, thereby promoting the differentiation of NSCs into motor neurons and the sprouting of neurites (Yao S. et al., 2024). This facilitates the repair of the injured region. For enhanced repair, conductive hydrogels incorporating capacitance-coupled in situ electrical stimulation can be implanted at the injury site, directly and effectively promoting neural tissue regeneration (Wu et al., 2024). In summary, conductive hydrogels modulate microglial polarization, astrocyte differentiation, and neuronal development, thereby improving the local environment and promoting axonal regeneration.

#### 4.2.2 Advances in the combined therapy of magnetic stimulation and biomaterials for axonal regeneration

Magnetic stimulation, characterized by its painless, non-invasive, and deep-penetrating properties, is emerging as a promising approach for promoting axonal regeneration in the CNS (Yang et al., 2023). By leveraging residual nerve fibers in the injured area, it facilitates the formation of complete neural pathways. Studies have demonstrated that nerve root magnetic stimulation enhances the recovery of synaptic ultrastructure in the sensory-motor cortex. Transcranial magnetic stimulation (TMS) maximally activates sensory-motor circuits, inhibits astrocyte differentiation, and reduces the expression of neuroinflammatory factors. Non-invasive supra-threshold high-frequency repetitive transcranial magnetic stimulation (HF-rTMS) promotes axonal regeneration and sprouting of the corticospinal tract, thereby achieving therapeutic effects for SCI (Boato et al., 2023). Furthermore, when combined with nanoparticles and hydrogels under magnetic mechanical stimulation, this approach fosters the differentiation of NSCs into functional neurons within an optimized microenvironment, further enhancing axonal regeneration (Zhang W. et al., 2024).

Electrical and mechanical stimulation therapy can modulate the microenvironment following SCI and enhance the plasticity of neural circuits and synapses. However, this approach may also elicit unknown biological responses (Usmani et al., 2020), and the potential side effects remain to be fully characterized. The optimal intensity of different stimuli requires further experimental investigation. Additionally, the intensity used in clinical applications varies among individuals. Ensuring adequate stimulation when the intensity is insufficient necessitates the use of complementary monitoring techniques to guide therapeutic decisions.

## 5 Conclusion

Axonal regeneration serves as the cornerstone for functional recovery following SCI. Acting as a bridge, it reconnects intact neural circuits and restores nerve conduction in the injured area, thereby facilitating post-injury functional recovery. The repair process not only depends on intrinsic growth mechanisms but also requires support from an optimal external microenvironment. This article reviews how enhancing microtubule stability, promoting actin filament disassembly, and upregulating axonal regeneration-associated proteins can stimulate the intrinsic growth of axonal growth cones. However, such intrinsic growth necessitates a supportive microenvironment. Restoring mitochondrial function provides energy for axonal regeneration; vascular reconstruction supplies essential nutrients; modulating inflammatory responses and glial scar formation minimizes barriers to regeneration. With advancements in biotechnological engineering, challenges related to targeted delivery and treatment have been addressed, thereby optimizing the pathways for achieving axonal regeneration. Secondary injury following spinal cord injury is a multifactorial and multipathway process. This article focuses on selected representative mechanisms, while further exploration of additional factors will be required in future studies. Future research should focus on developing biomaterials that closely mimic normal tissue, better integrating with the injury site’s structure and providing therapeutic benefits. When combined with physical stimulation, these biomaterials can synergistically improve the pathological microenvironment, promote axonal regeneration, establish new neural circuits, and ultimately facilitate functional recovery.
